# Evaluation of Clinical and Immunopathological Features of Different Infective Doses of *Trypanosoma cruzi* in Dogs during the Acute Phase

**DOI:** 10.1100/2012/635169

**Published:** 2012-04-01

**Authors:** Israel A. Quijano-Hernández, Alejandro Castro-Barcena, Esteban Aparicio-Burgos, Marco A. Barbosa-Mireles, Julio V. Cruz-Chan, Juan C. Vázquez-Chagoyán, Manuel E. Bolio-González, Eric Dumonteil

**Affiliations:** ^1^Facultad de Medicina Veterinaria y Zootecnia, Universidad Autónoma del Estado de México, Cerrillo Piedras Blancas s/n, 50200 Toluca, Mexico; ^2^Laboratorio de Parasitología, Centro de Investigaciones Regionales “Hideyo Noguchi”, Universidad Autónoma de Yucatán, 97000 Mérida, Mexico; ^3^Facultad de Medicina Veterinaria y Zootecnia, Universidad Autónoma de Yucatán, Carretera Mérida-Xmatkuil, km 15, 97315 Mérida, Mexico

## Abstract

Infection with *Trypanosoma cruzi* is a major risk in Latin America, and dogs are believed to be good models for evaluating Chagas disease. Here, we evaluated the clinical and immunopathological alterations developed by mongrel dogs experimentally infected with different infective doses (2,000, 20,000, and 200,000 metacyclic trypomastigotes of Sylvio X10/4 strain kg^−1^ via intraperitoneal). Clinical and electrocardiographic parameters, as well as antibody production and pathologic lesions were evaluated. All three doses of this strain of *T. cruzi* induced a similar pattern of infection characterized by cardiac arrhythmias and severe and diffuse myocarditis. Specific anti-*T. cruzi* IgG indicated seroconversion by day 14 after infection, and IgG levels increased during the period of evaluation. Mortality was observed only in dogs infected with the medium or high parasite doses, but not in the group infected with a low dose of 2,000 parasites kg^−1^. Infection with a low dose of parasites provides an excellent nonlethal model to evaluate the immunopathology of the acute disease in dogs infected with the Sylvio X10/4 strain of *T. cruzi*.

## 1. Introduction

Chagas disease is a major public health threat in Latin America, and dogs are an important domestic reservoir for infection in humans [1]. In Argentina, the presence of dogs in dwellings increases the risk for human infection about 3–5 times [2].

Dogs have also been used as a model for evaluating the process of disease development following *Trypanosoma cruzi* infection [1, 3, 4]. Several studies have reported the use of different strains and doses to evaluate the infection, which makes difficult to correlate and compare the results between them. Strains such as *Berenice 78* [5, 6], SC-1 [7] or* Tc-O *[8] have been used, with doses ranging from one thousand up to 5 million trypomastigotes per kilogram of body weight. It has been demonstrated that different strains, routes of inoculation and animal species developed different degrees of lesion and immunological response and infection affected a variety of organs [9].

Indeed,* T. cruz*i parasites have variable tropisms to different organs, which has been attributed to the strain, number of parasites, the origin of trypomastigotes, the route of infection, among others [10, 11]. Other studies have compared blood and metacyclic trypomastigotes, and the latter were found to be more pathogenic since they produced a more severe form of disease [6].

In order to evaluate the effect of infection by different parasite doses on the immunopathology of *T. cruzi *infection in dogs, we evaluated here three different doses of metacyclic trypomastigotes of the Sylvio X10/4 strain, considered a pathogenic strain [9], administered via intraperitoneal, to determine the best challenge model to further study the immunopathology of *T. cruzi* in this species.

## 2. Materials and Methods

### 2.1. Dogs

Eleven clinically healthy mongrel dogs ranging from 4 to 6 months of age, without clinical and electrocardiographic (ECG) alterations seronegative for *T. cruzi* IgG by ELISA, were used. They were dewormed, and vaccinated twice against canine distemper virus, canine parvovirus, parainfluenza, leptospira, and adenovirus type 2, and fed with a balanced commercial food. The dogs were numbered and handled as described by the official protocols of NOM-062-ZOO-1999. All procedures were carried out in compliance with the Ethical committee for animal welfare of the Center of Advanced Studies on Animal Health of the Faculty of Veterinary Medicine of the Autonomous University of Mexico State.

### 2.2. Parasites and Inoculums

Sylvio X10/4 *T. cruzi* strain, kindly donated by Dr Nisha Garg (University of Texas Medical Branch, Galveston), was maintained by serial passes in NIH 3T3 cells in DMEM medium with 10% fetal bovine serum (Hyclone, USA), pH 6.8, 5% CO_2_ at 37°C [12]. The dogs (D) were randomly assigned into one of three groups to be infected with 200,000 (high dose) (D1–D3), 20,000 (medium dose) (D4–D7) or 2,000 (low dose) (D8–D11)-live metacyclic trypomastigotes kg^−1^.

### 2.3. Clinical Evaluation

After infection, dogs were evaluated clinically for signs of disease such as fever, depression, and anorexia on a daily basis. Hemograms and manual differential counts were performed every two weeks using an *AcT *Coulter programmed for veterinary species according to equipment instructions. Body weight was measured weekly. Mortality was calculated for each group.

### 2.4. Electrocardiography

Electrocardiographic (ECG) recordings were performed at a 50 mm/s and 1 mV/cm sensitivity and bipolar and unipolar leads were recorded, with a Cardiofax, NIHON-KOHDEN [13], every two weeks after infection (PI) [14].

### 2.5. Serology

Blood samples were obtained by venipuncture of cephalic vein every two weeks, and total IgG was determined using MicroELISA (Laboratorio Lemos SRL, Buenos Aires Argentina), following the manufacturer's indications, except that a horse-radish-peroxidase- (HRP-) labeled goat anti-dog IgG (Bethyl Laboratories) was used at 1 : 7500 [15]. Dogs serum was used at a 1 : 100 dilution and the cut-off value (OD 450 nm + 2 SD) was set using sera from 30 seronegative dogs.

### 2.6. Parasitemia

Five microliters of EDTA venous blood were collected every other day starting from day seven until day 45, and parasites were counted [9]. Briefly, 5 *μ*L of blood was directly placed between a slide and a cover slide 18 × 18 mm and parasites were counted; then results were multiplied by 200 to obtain the number of trypomastigotes per milliliter.

### 2.7. Pathology and Histopathology

At day 45 after infection, the dogs were euthanized by barbiturates overdose. During necropsy hearts were removed and weighted. Heart weight (g) to body weight (kg) ratio was calculated [1], to determine cardiomegaly and differences among groups. Myocardium sections of 0.5 cm^3^ from each wall (right and left auricle and ventricles and interventricular wall) were cut and embedded in paraffin; then, 5-6 *μ*m thick sections cuts were made from each wall and stained with hematoxylin and eosin to evaluate myocarditis in each dog. Ten randomly selected fields at 400x were evaluated for inflammation using scores **0**: when no inflammation was demonstrated; **1**: when 30% or less of a field at 400x was infiltrated; **2**: when 31–60% was affected, and **3**: when more than 65% of a field was occupied by inflammatory cells; and differences between cardiac walls and groups were evaluated [14]. Amastigote pseudocysts were counted in 50 randomly selected fields.

### 2.8. Statistical Analyses

ECG alterations, mortality, cardiac inflammation, and hematological variables were analyzed with nonparametric statistics (Wilcoxon, Kruskall-Wallis and Dunn's post hoc test) due to their nonnormal distribution and categorical value for some results. All statistical analyses were performed using Prism 4.0 (Graph Pad, CA, USA).

## 3. Results

### 3.1. Clinical Observations

#### 3.1.1. Clinical Evaluation

All animals gained weight continuously until the end of the experiment, and no weight differences were observed among groups. No clinical signs of disease or cardiopathy were displayed by dogs during the first 3 weeks of infection; though a dog from the medium dose group died by day 25 after infection and a dog from the high dose group died by day 36, both from cardiomyopathy as shown by necropsy and histopathology. Mortality was thus observed in 1/3 (33%) animals infected with a high parasite dose, 1/4 (25%) from the group infected with a medium parasite dose, and 0/4 (0%) in the group infected with a low parasite dose.

#### 3.1.2. Hematologic Findings

Dogs from all groups maintained their hematocrit within the reference interval (0.37–0.54 L/L), but developed eosinophilia (>1.3 × 10^9^/L) by week 1 after infection, which lasted until the third week, though no statistical differences were found between groups (*P* > 0.05). Lymphocyte counts were significantly higher on day 14 after infection in the dogs of the high-compared to the low dose group (*P* < 0.01), but no statistical differences were found between these groups and the medium dose group. No differences were found among groups in lymphocyte counts in samples collected on any other day (days 0, 28, and 42).

#### 3.1.3. Electrocardiography

Dogs from every group developed electrocardiographic alterations such as right bundle branch block (RBBb) (D2 day 14, D8 day 42), low sensitivity of QRS complex (D1 day 28, D5 day 28), and Atrioventricular block (D10 and D11 at day 28), and D2 also developed Ventricular tachycardia considered a life-threatening arrhythmia if not treated (Table 1).

### 3.2. Serology

**Figure 1:**
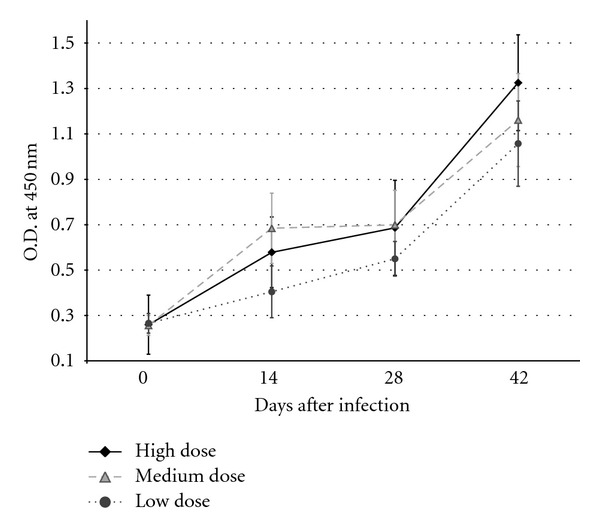
Total IgG levels along the evaluation period. Mean and S.D. of OD_450 nm_ for groups infected with high (200,000 MT), medium (20,000 MT) and low (2,000 MT) parasite doses, showing a constant increase in antibody levels in dogs on days 0, 14, 28 and 42 post infection. No statistical differences were found between groups (*P* > 0.05).

Following infection, all dogs became seropositive by the second week after infection by ELISA IgG test, except one dog from the medium dose group, which seroconverted at week 4. Antibody levels increased constantly during the follow-up until the end of the experiment, but no statistical difference was observed between groups (*P* > 0.05, Figure 1).

### 3.3. Parasitology

**Figure 2:**
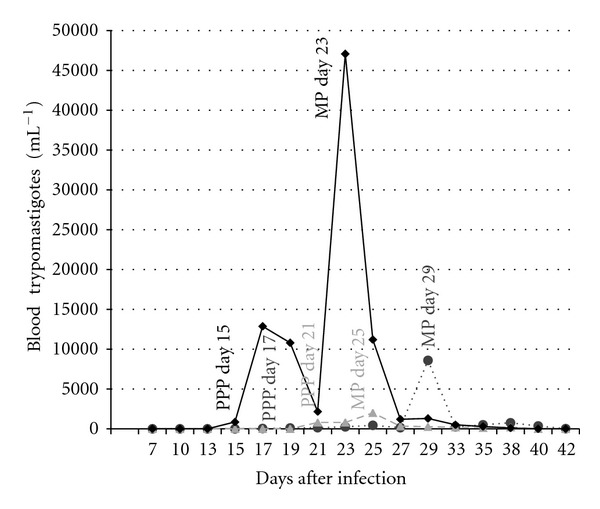
Parasitemia. Mean parasitemia at different days postinfection, showing day of prepatent period (PPP), maximum peak of parasitemia (MP), showing a very erratic pattern alternating negative with positive parasitemia readings. All dogs showed parasitemia but no differences were found among groups (*P* > 0.05). Same symbols as Figure 1.

All dogs developed a low parasitemia (Figure 2), except one dog from the high parasite dose group, which displayed a high peak of parasitemia (47,000 blood trypomastigotes mL^−1^) on day 23. Mean prepatent period lasted 15, 17, and 21 days for dogs infected with a high, medium, and low parasite dose, respectively. The peak of parasitemia was found on days 23, 29, and 25, and lasted for 13, 11, and 21 days for these groups, respectively. Significant differences were not found for the prepatent period, the patent period, nor for the peak of parasitemia due to the large variability among individual dogs (Figure 2).

Amastigote nests were present in cardiac tissue of all dogs, except D6, and were found more frequently in right ventricular tissue.

### 3.4. Pathology

**Figure 3:**
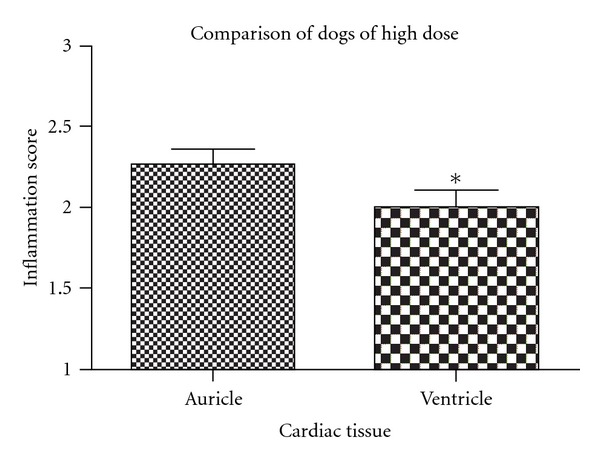
Auricular tissue presents significantly more inflammation than ventricular (*P* = 0.0027). Mean + SEM is shown.

**Figure 4:**
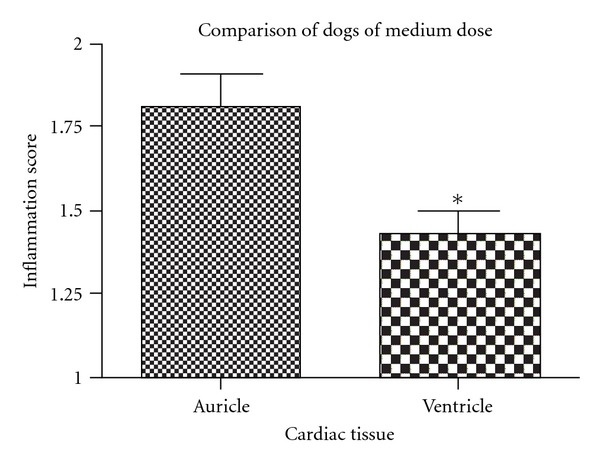
Auricular tissue also presents significantly more inflammation than ventricular at this dose (*P* = 0.0032) and Mean + SEM is shown.

At necropsy, all animals displayed slight right ventricle dilation and infarcted areas distributed all over the cardiac tissue. Heart:body weight ratio (Cardiac index) was not significantly different among groups. Microscopically, pseudocysts were found in all dogs from the high-dose group and in one dog from the medium-dose group. No differences were found among auricular and ventricular walls or septum tissue in the dogs from the low-parasite-dose group. However, a larger inflammation score was found in auricles compared to ventricles for dogs infected with a medium dose (*P* = 0.027, Figure 3) and with a high dose, respectively (*P* = 0.0032, Figure 4).

**Figure 5:**
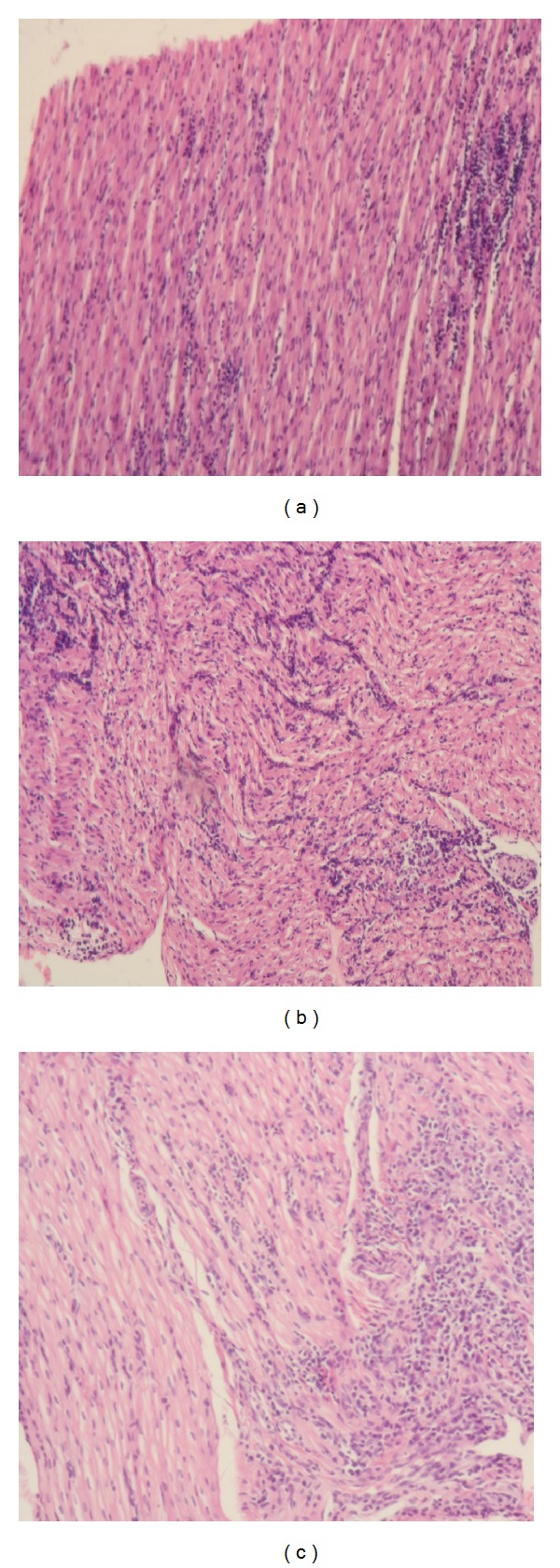
(a) Slight myocarditis in ventricular wall of dog 9 of the low dose group. (b) Lymphoplasmacytic myocarditis in ventricular wall of dog 2 of the high dose group. (c) Ventricular wall of dog 4 of medium dose group. Even a dose as low as 2000 trypomastigotes per kilogram of *T. cruzi *strain Sylvio 10x can produce evident lesions in myocardium of dogs.

Different degrees of lymphoplasmacytic inflammation were found in the myocardium of dogs of each different group (Figure 5).

## 4. Discussion

Although several studies have been performed to test different protocols of experimental infection with infective forms of *T. cruzi*, there is a lack of standardization on the route, stage or number of parasites used. Some authors report the use of blood trypomastigotes, which are easier to obtain by serial passes in mice [14], while others report the use of metacyclic trypomastigotes [1, 3, 6], which are more appropriate because this is the form through which natural infection occurs. Still, other authors have tested differences in infection using different routes of inoculation than the intraperitoneal route. The clinical, hematological, parasitological, immunological and pathological variables are not systematically analyzed in the different studies, which makes it more difficult to compare their results. Standardizing the model might improve this comparison.

In the present study dogs were infected with one of three different doses of parasites (200,000, 20,000, or 2,000 metacyclic trypomastigotes kg^−1^). During the first 3 weeks after infection, no clinical signs of disease such as fever, anorexia, pain or diarrhea were found during clinical inspection. The absence of signs has been previously reported when animals were infected with different stage forms of *Trypanosoma cruzi*, it has been observed in natural infections possibly because the infection may be well controlled in some animals, though others may show severe cardiac insufficiency due to lethal myocarditis [1, 14]. These findings are in agreement with the clinical difficulty to detect the acute phase of the disease in naturally occurring infection during a routine clinical inspection, particularly if we consider that natural infections are likely to occur with an even lower number of parasites. In the present study, despite the absence of such clinical signs, one dog infected with a medium dose and one infected with a high dose died suddenly, on days 25 and 36 after infection, respectively. Both animals died from cardiomyopathy as was demonstrated by necropsy and histopathology and both animals presented abnormal electrocardiographic readings on days 14 or 28. Such abnormalities included severe arrhythmias (RBBb and VT). Surprisingly, dogs with the lowest parasite dose displayed more arrhythmias (RBBb, AVb I, and AVb I) than those from the groups with higher infection doses. These forms of arrhythmias have been reported during natural infection in previous studies [6, 8, 16]. These findings show that ECG recordings are a good parameter to evaluate the progress of disease even at low doses of inoculum. Eosinophilia was the most frequent finding in hemograms, which support the idea that eosinophilia might be a nonspecific hallmark for the acute phase of *T. cruzi* infection [13]. Higher lymphocytosis was observed at day 14 after infection in dogs infected with a high parasite dose. Hematological alterations such as eosinophilia, lymphocytosis, and anemia were observed in all dogs of our study, confirming the effectiveness of infection.

Anti-*T. cruzi* IgG antibody levels increased continuously during the experimental period, thus, this parameter may be a good indicator for the development of the immunologic response that can be used to monitor infection development. Metacyclic trypomastigotes have been shown to promote a higher production of IgG antibodies than blood trypomastigotes [6]. Total IgG serum levels were not affected by the number of parasites inoculated, however, as no differences could be observed on days 14, 28, and 45 post-infection. This may be due to an effective activation of B cells that produce high levels of IgG independently of the number of parasites inoculated. Infection was confirmed as parasitemia was observed in all dogs; however, levels of parasitemia were erratic, alternating negative to high counts of blood trypomastigotes. This variability led to large standard deviations, and therefore no statistical differences could be found among treatments. This finding supports the idea that parasitemia is not a very reliable diagnostic parameter used as a single diagnostic test if a single day is evaluated during the acute phase of the disease, and could be explained based on individual factors such as immunity and genetics, as it has been proposed in murine models [4]. The infection with the lower parasite dose resulted in a delayed peak of parasitemia (almost a week later) compared with the high-dose group.

Amastigote nests were present in cardiac tissue of all dogs except of dog no. 6, and the lowest infectious dose was able to produce amastigote nests. The right ventricle was the more frequent location for amastigote nests and might be the tissue of election to evaluate parasitic load; therefore quantitative real-time PCR from heart tissue would be an adequate test to compare parasitic load in this organ, in experimentally infected animals. No differences were found among groups on heart : body weight ratio (cardiac index), likely because cardiomegaly requires a longer time period to develop, as suggested previously [13]. While evaluating the intensity of inflammation of myocardium, it was found that auricles were more severely affected than ventricles in dogs from the high- and medium-dose but not in dogs from the low-dose group. In addition, the auricle within the same dog was systematically the most affected wall with the highest inflammation score. These findings are in contrast with previous observations [14], in which the right ventricle was the most affected using a local strain of *T. cruzi* from Yucatán, México. It is interesting to note that, even though the auricle displayed the most severe inflammatory reaction, amastigotes were most frequently found in the wall of the right ventricle. These findings may indicate that the primary invasion could have occurred in auricular tissue, followed by a severe inflammatory response that had been working toward elimination of parasites earlier than other cardiac tissue areas, which could explain why auricles had lower numbers of amastigote nests compared with ventricular tissue, which could have been infected at a later time with the consequent delayed inflammatory response.

## 5. Conclusions

Our dog model and the infective dose of 2,000 metacyclic trypomastigotes kg-1 of the Sylvio X10 strain of *Trypanosoma cruzi* reproduces the main clinical and pathological features without being lethal during the course of the first 45 days (acute phase) in dogs, as confirmed by the presence of parasites, cardiac arrhythmias, and myocarditis after a short follow-up period. Such standardization of an experimental challenge capable of inducing the disease is considered essential when evaluating drugs or vaccines. Serological, clinical, and standard histopathological methods used in the present study were able to follow-up infection at all doses, although parasitemia was highly variable. ELISA is widely used for the diagnosis of *T. cruzi* infection because it is sensitive with good specificity. In the present study this diagnostic tests determined infection in dogs very sensitively at any inoculation dose. This model provides the basis for future studies concerning immunity and pathology and may be relevant to study therapeutic agents as well as vaccines.

##  Authors' Contributions

All authors contributed equally to this work. I. A. Quijano-Hernández, J. V. Cruz-Chan, and E. Dumonteil contributed by study conception, design, and data analysis; I. A. Quijano-Hernández, A. Castro-Barcena, E. Aparicio-Burgos, and M. Barbosa-Mireles by data adquisition, analysis, and interpretation; I. A. Quijano-Hernández, J. V. Cruz-Chan, M. Bolio-González, and E. Dumonteil by drafting and revision of the paper. All authors finally approved the paper.

##  Conflict of Interests

The authors declare that there is no conflict of interests.

## Figures and Tables

**Table 1:** Electrocardiography follow-up during evaluation period.

Day	0	14	28	42
Group of high dose (200,000 MT)				

D1	Normal	Normal	Low sensitivity QRS complex	Normal
D2	Normal	RBBB	Ventricular tachycardia	**+**
D3	Normal	Normal	Normal	Normal

Group of medium dose (20,000 MT)				

D4	Normal	Normal	Normal	Normal
D5	Normal	Normal	Low sensitivity QRS complex	**+**
D6	Normal	Normal	Normal	Normal
D7	Normal	Normal	Normal	Normal

Group of low dose (2,000 MT)				

D8	Normal	Normal	Normal	RBBB
D9	Normal	Normal	Normal	Normal
D10	Normal	Normal	AVB I	AVB I
D11	Normal	Normal	AVB I	AVB I

Development of EKG alterations in the dogs of different groups. Two dogs of low-dose-developed EKG alterations but these were mild and not life threatening in contrast to those developed by dogs of group high-dose and medium-dose groups which were fatal. AVB I: Atrioventricular block grade 1; RBBB: right bundle branch block BRD; + dog dead.

## References

[1] Guedes P, Veloso V, Caliari M (2007). *Trypanosoma cruzi* high infectivity in vitro is related to cardiac lesions during long-term infection in Beagle dogs. *Memorias do Instituto Oswaldo Cruz*.

[2] Gürtler R, Chuit R, Cécere M, Castañera M, Cohen J, Segura E (1998). Household prevalence of seropositivity for *Trypanosoma cruzi* in three rural villages in northwest Argentina: environmental, demographic, and entomologic associations. *The American Journal of Tropical Medicine and Hygiene*.

[3] De Lana M, Chiari E, Tafuri W (1992). Experimental Chagas’ disease in dogs. *Memorias do Instituto Oswaldo Cruz*.

[4] Andrade ZA (1999). Immunopathology of Chagas disease. *Memorias do Instituto Oswaldo Cruz*.

[5] Gürtler R, Ceballos L, Ordóñez-Krasnowski P, Lanati L, Stariolo R, Kitron U (2009). Strong host-feeding preferences of the vector *Triatoma infestans* modified by vector density: implications for the epidemiology of Chagas disease. *PLoS Neglected Tropical Diseases*.

[6] Carneiro M, Martins-Filho O, Reis A (2007). Differential impact of metacyclic and blood trypomastigotes on parasitological, serological and phenotypic features triggered during acute *Trypanosoma cruzi* infection in dogs. *Acta Tropica*.

[7] Machado E, Fernandes AJ, Murta S (2001). A study of experimental reinfection by *Trypanosoma cruzi* in dogs. *The American Journal of Tropical Medicine and Hygiene*.

[8] Morris S, Barr S, Weiss L, Tanowitz H, Wittner M, Bilezikian J (1991). Myocardial *β*-adrenergic adenylate cyclase complex in a canine model of chagasic cardiomyopathy. *Circulation Research*.

[9] Marinho C, Bucci D, Dagli ML (2004). Pathology affects different organs in two mouse strains chronically infected by a *Trypanosoma cruzi* clone: a model for genetic studies of Chagas’ disease. *Infection and Immunity*.

[10] Brener Z (1985). General review on *Trypanosoma cruzi* classification and taxonomy. *Revista da Sociedade Brasileira de Medicina Tropical*.

[11] Noireau F, Diosque P, Jansen M (2009). *Trypanosoma cruzi*: adaptation to its vectors and its hosts. *Veterinary Research*.

[12] Manning-Cela R, Cortés A, González-Rey E, Wesley CV, Swindle J, González A (2001). LYT1 protein is required for efficient in vitro infection by *Trypanosoma cruzi*. *Infection and Immunity*.

[13] Cruz-Chan J, Bolio-González M, Colín-Flores R, Ramirez-Sierra M, Quijano-Hernández I, Dumonteil E (2009). Immunopathology of natural infection with *Trypanosoma cruzi* in dogs. *Veterinary Parasitology*.

[14] Quijano-Hernandez I, Bolio-González M, Rodríguez-Buenfil J, Ramírez-Sierra M, Dumonteil E (2008). Therapeutic DNA vaccine against *Trypanosoma cruzi* infection in dogs: a pilot clinical trial. *Annals of the New York Academy of Sciences*.

[15] Barbabosa-Pliego A, Díaz-Albiter HM, Ochoa-García L (2009). *Trypanosoma cruzi* circulating in the southern region of the State of Mexico (Zumpahuacan) are pathogenic: a dog model. *The American Journal of Tropical Medicine and Hygiene*.

[16] Basso B, Castro I, Introini V, Gil P, Truyens C, Moretti E (2007). Vaccination with Trypanosoma rangeli reduces the infectiousness of dogs experimentally infected with *T. cruzi*. *Vaccine*.

